# An Environmental Contributor to Parkinson’s Disease Causes a Hormetic Lifespan Effect in C. elegans

**DOI:** 10.1093/geroni/igab046.3358

**Published:** 2021-12-17

**Authors:** Jennifer Thies, Hanna Kim, Guy A Caldwell, Kim A Caldwell

**Affiliations:** The University of Alabama, Tuscaloosa, Alabama, United States

## Abstract

Only 5-10% of Parkinson’s Disease (PD) cases have a direct genetic origin; however, exposure to herbicides, pesticides, and interactions with soil are potential risk factors. PD is characterized by the loss of dopaminergic (DA) neurons and the formation of protein inclusions that contain α-synuclein (α-syn). Conversely, a soil bacterium, Streptomyces venezuelae (S. ven), produces a secondary metabolite that causes age- and dose- dependent DA neurodegeneration in C. elegans; it also exacerbates α-syn-induced DA neurodegeneration. Previous studies from our lab determined that exposure to the S. ven metabolite caused oxidative stress, mitochondrial fragmentation and enhanced reactive oxygen species (ROS). Here we report that exposure to S. ven metabolite causes a hormetic effect on C. elegans lifespan, where low concentrations (5X) extend lifespan in N2 animals, but at higher concentrations (20X) lifespan is decreased. To further examine this hormetic response, we examined daf-16 mutants in this assay. daf-16 mutants displayed no significant differences between solvent and metabolite at both high and low concentrations, suggesting the hormetic response is daf-16 dependent. We also studied S. ven metabolite on C. elegans aging mutants. We investigated mutants in the AMPK signaling pathway and found when exposed to the 20X concentration of S. ven metabolite, aak-2 mutants displayed no significant difference between solvent and metabolite over lifespan. However, when aak-2 mutants were exposed to solvent control and the 5X concentration, mutants displayed a decreased lifespan. This suggests that functional aak-2 might be important for increased lifespan when combating toxicants following chronic exposure.

